# A nomogram combining thoracic CT and tumor markers to predict the malignant grade of pulmonary nodules ≤3 cm in diameter

**DOI:** 10.3389/fonc.2023.1196883

**Published:** 2023-06-08

**Authors:** Jianhao Qiu, Rongyang Li, Yukai Wang, Xiuyuan Ma, Chenghao Qu, Binyan Liu, Weiming Yue, Hui Tian

**Affiliations:** ^1^ Department of Thoracic Surgery, Qilu Hospital of Shandong University, Jinan, Shandong, China; ^2^ Department of Cardiology, Qianfoshan Hospital in the Shandong Province, Jinan, Shandong, China; ^3^ Department of Breast Surgery, Qilu Hospital of Shandong University, Jinan, Shandong, China

**Keywords:** pulmonary nodules, consolidation tumor ratio, CT values, carcinoembryonic antigen, nomogram

## Abstract

**Background:**

With the popularity of computed tomography (CT) of the thorax, the rate of diagnosis for patients with early-stage lung cancer has increased. However, distinguishing high-risk pulmonary nodules (HRPNs) from low-risk pulmonary nodules (LRPNs) before surgery remains challenging.

**Methods:**

A retrospective analysis was performed on 1064 patients with pulmonary nodules (PNs) admitted to the Qilu Hospital of Shandong University from April to December 2021. Randomization of all eligible patients to either the training or validation cohort was performed in a 3:1 ratio. Eighty-three PNs patients who visited Qianfoshan Hospital in the Shandong Province from January through April of 2022 were included as an external validation. Univariable and multivariable logistic regression (forward stepwise regression) were used to identify independent risk factors, and a predictive model and dynamic web nomogram were constructed by integrating these risk factors.

**Results:**

A total of 895 patients were included, with an incidence of HRPNs of 47.3% (423/895). Logistic regression analysis identified four independent risk factors: the size, consolidation tumor ratio, CT value of PNs, and carcinoembryonic antigen levels in blood. The area under the ROC curves was 0.895, 0.936, and 0.812 for the training, internal validation, and external validation cohorts, respectively. The Hosmer-Lemeshow test demonstrated excellent calibration capability, and the fit of the calibration curve was good. DCA has shown the nomogram to be clinically useful.

**Conclusion:**

The nomogram performed well in predicting the likelihood of HRPNs. In addition, it identified HRPNs in patients with PNs, achieved accurate treatment with HRPNs, and is expected to promote their rapid recovery.

## Introduction

Lung cancer is one of the leading causes of cancer related deaths worldwide with a 5-year overall survival (OS) rate of 19% (1). The popularity and development of low-dose thorax computed tomography (CT) have increased the diagnosis for patients with early-stage lung cancer (2). Pulmonary nodules (PNs) are early imaging manifestations of lung cancer, which are defined as focal, round-like, subsolid, or solid lung shadows with a diameter ≤3 cm (3, 4). PNs can be classified into a variety of pathological types, of which adenocarcinoma is the most common histological type; squamous cell and neuroendocrine carcinoma may also occur. Based on the fifth edition of the World Health Organization classification of thoracic tumors, atypical adenomatoid hyperplasia (AAH) and adenocarcinoma *in situ* (AIS) are classified as glandular precursor lesions. Adenocarcinoma is divided into two subtypes: minimally invasive adenocarcinoma (MIA) and invasive adenocarcinoma (IAC) (5, 6). Existing studies have demonstrated that radical resection can be achieved in both AIS and MIA patients. Among them, the 10-year OS rate was as high as 100% in AIS patients, while the 10-year OS rate in MIA patients was not significantly different from that of AIS (97.8%) (7, 8). For patients with IAC, however, the prognosis of early-stage Ia lung cancer is highly variable due to the different pathological subtypes. For lepidic invasive adenocarcinoma, the 5-year OS can reach 86%-100%, whereas the 5-year OS for solid invasive adenocarcinoma (SPA) and micropapillary invasive adenocarcinoma (MPA) is less than 60% (9–11). Therefore, ensuring that high-risk pulmonary nodules (HRPNs) be promptly resected at an early stage while avoiding overtreatment of low- risk pulmonary nodules (LRPNs) is an urgent need.

Histopathology remains the gold standard for a diagnosis of lung cancer (12, 13); however, given the invasive nature of surgery, the preoperative malignancy of early-stage PNs is not highly valued. The detection of tumor markers, such as carcinoembryonic antigen (CEA), cytokeratin fragment antigen 21-1 (CYFRA21-1), squamous cell carcinoma antigen (SCC), progastrin-releasing peptide (Pro-GRP), carbohydrate antigen 125 (CA125), and neuron-specific enolase (NSE), for lung cancer is widely used in clinical practice (14–17); however, it can easily cause errors in clinical diagnosis owing to its relatively low specificity. Thoracic CT is useful for diagnosing lung cancer. Previous studies have demonstrated that the consolidation tumor ratio (CTR) of PNs can be a critical parameter to predict the degree of malignancy and to determine the surgical method with a specificity of 98.7% (18). However, since early-stage lung cancers with different invasive degrees have overlapping CT morphologies, the CTR of the same PNs measured by different radiologists may be subjective to some extent, affecting the risk assessment of early-stage PNs. Accurately predicting the malignancy of patients’ PNs without invasive procedures remains a considerable challenge for clinicians, and there is a need to construct an effective model for preoperative prediction of the risk for PNs with diameter ≤3 cm.

As an emerging visual statistical prediction model, a nomogram is often used to quantify the risk of clinical events, such as cancer (19, 20). No nomogram has incorporated CT imaging features and lung cancer tumor markers to differentiate HRPNs from LRPNs accurately. Therefore, our goal was to develop a nomogram based on important demographic information, clinical parameters, CT imaging characteristics, and tumor markers to assess the risk of PNs ≤3 cm in diameter prior to treatment and assist thoracic surgeons in making clinical decisions.

## Patients and methods

### Protocol and ethics statement

This retrospective study was conducted in accordance with the principles of the Declaration of Helsinki, and the Ethics Committee of Qilu Hospital of Shandong University approved the protocol (registration number: KYLL- 202008-023-1). All patients gave informed consent for the use of their clinical data.

### Patients’ selection

A retrospective analysis of 1064 patients hospitalized for PNs in Qilu Hospital of Shandong University from April to December 2021 was conducted by searching a prospectively maintained database. The exclusion criteria were as follows: (i) age <18 years, (ii) no thoracic CT within two week before surgery, (iii) preoperative puncture biopsy or fiberoptic bronchoscopy confirmed lung malignancy, (iv) multiple pulmonary nodules, (v) postoperative pathology of pulmonary metastatic tumors, and (vi) incomplete perioperative data. Overall, 812 patients with 812 lesions met the inclusion criteria and were randomly allocated to either the training or internal validation cohort in a 3:1 ratio according to the random split-sample method. In addition, following the inclusion criteria, the thoracic surgery database of Qianfoshan Hospital of Shandong Province was searched, and 83 patients treated at this hospital from January to April 2022 were included as an independent external validation cohort. The training cohort was used to develop a predictive nomogram, while the validation cohorts were used to validate the nomogram’s performance.

### CT scanning

Before the surgical intervention, all patients underwent a thoracic CT scan (Brilliance iCT 256, Philips Healthcare, USA). The CT imaging parameters were as follows: tube voltage, 120 kV; tube current, 150 mA; field of view, 350 mm; slice thickness, 1.0 mm; and gantry rotation time, 270 ms.

### Image assessment

**Figure 1:**
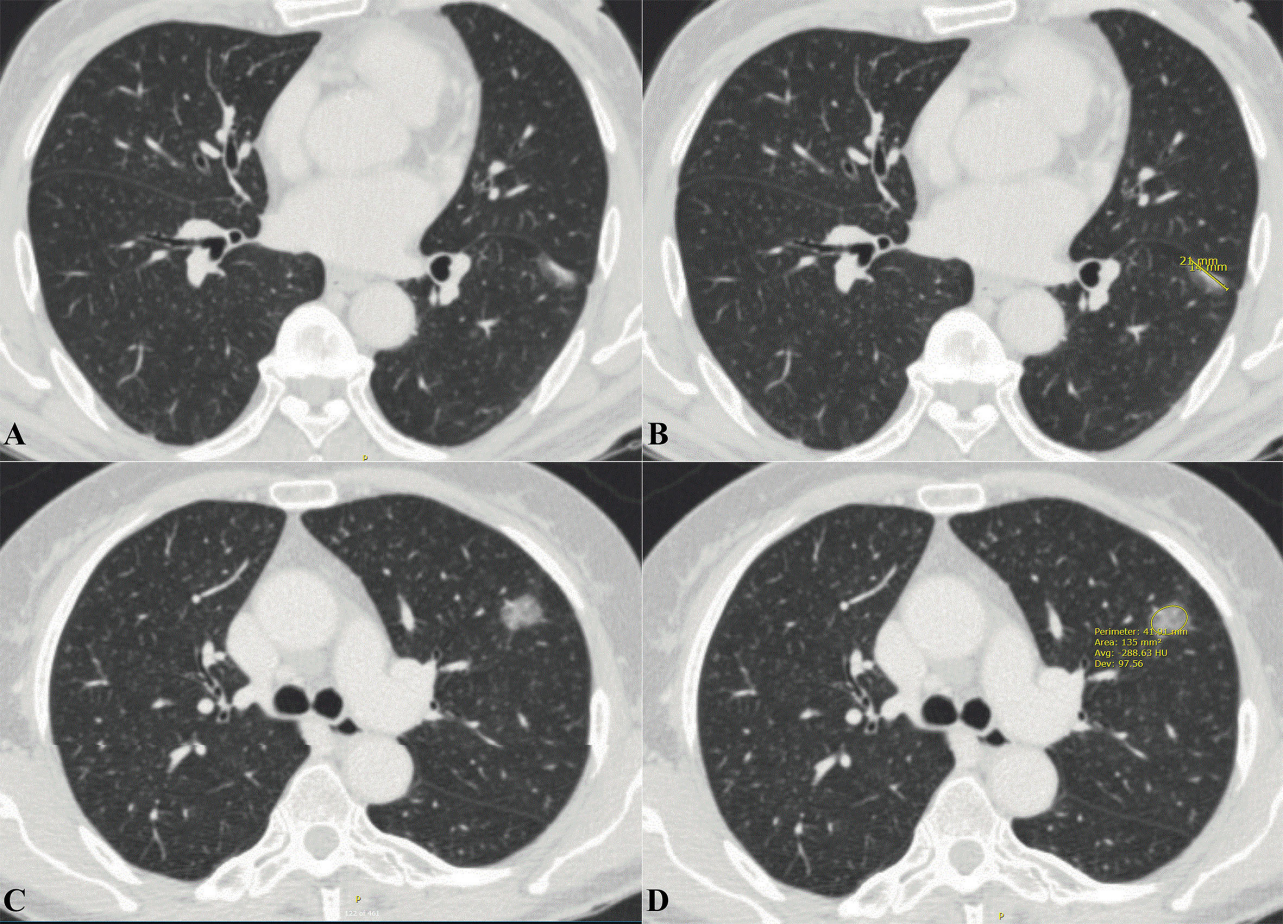
Extraction of imaging data related to pulmonary nodules from thoracic CT. **(A)** Axial thoracic CT images showed a mixed density ground-glass nodule in the left lower lobe. Histology confirmed it as IAC. **(B)** The long diameter of the solid component was measured to be 14mm and the long diameter of the whole pulmonary nodule to be 21mm. The CTR value was about 0.677. **(C)** Axial thoracic CT images showed a high-density ground-glass nodule in the left upper lobe. Histology confirmed it as IAC. **(D)** Mean CT values of ground-glass nodule is -288.63 HU. CT, Computed tomography; IAC, Invasive adenocarcinoma; CTR, Consolidation tumor ratio.

The CT imaging features were independently assessed by two experienced thoracic surgeons (Jianhao Qiu and Rongyang Li) who were blinded to the patient’s clinical and pathological information prior to the assessment. Each lesion was assessed based on nodule diameter, CT values, CTR, lobulation, spiculation, bronchus-encapsulated air sign (BEAS), cavity sign, pleural traction, and vascular convergence. The nodule diameter was defined as the diameter (mm) of the largest lesion on an axial image at a given level (**Figure 1**). The CT values of the PNs were measured on the thoracic window with the largest nodule diameter using either a circular or oval region of interest, covering at least one-half of the nodule’s larger surface area, excluding obvious vessels and bronchi (**Figure 1**). The CT value measurement was repeated three times for all PNs, and the average CT values obtained six times by two thoracic surgeons were chosen as the representative PNs values. The CTR was defined as the ratio between the largest diameter of the solid component of the PNs and the largest diameter of the ground-glass nodule. For the irregularly shaped nodules, the average of the long and short diameters was used (**Figure 1**). Lobulation was defined as a portion of the surface of the lesion showing a scallop-like sign. Spiculation was defined as cords extending from the rim of the nodule into the lung parenchyma without reaching the pleural surface. BEAS was defined as small air-like low-density shadows with a smooth inner edge and a diameter <5 mm. Low-density shadows with a diameter ≥5 mm were defined as cavity signs. A linear, tentorial, or stellate shadow between the PNs and pleura was considered pleural traction. Vascular convergence was defined as a packing of the inner portion of the PNs or abnormal angulation to the PNs compared to that of normal lung parenchyma (**Supplementary Figures 1, 2**).

### Histopathologic features analysis

All pathological specimens were fixed in formalin, sectioned, and stained with hematoxylin-eosin, following standard pathological section preparation procedures. Light microscopic histopathological evaluation of hematoxylin-eosin- stained slides was performed by two experienced pathologists blinded to patient data, who independently evaluated each tissue section. Following the fifth edition of the World Health Organization classification of thoracic tumors, we classified PNs diagnosed as benign lesions, AAH, AIS, MIA, and well- differentiated lung cancer into the low-risk group. The pathological types of PNs classified as high-risk include: (i) moderately differentiated lung cancer; (ii) poorly differentiated lung cancer; (iii) well-differentiated lung adenocarcinoma with high-grade components accounting for ≥5%; and (iv) well-differentiated lung adenocarcinoma with high-risk factors. High-grade components include solid, micropapillary, cribriform, and complex glandular structures (fused glands or single cells infiltrating the desmoplastic stroma). High-risk factors include lymph node metastasis, neural invasion, visceral pleural invasion, vascular tumor thrombus, and airspace spread.

### Nomogram construction

To identify potential risk factors for HRPNs, univariable logistic regression analysis was conducted. Any factor with a P value of less than 0.2 in the univariable analysis was included in further multivariable analysis. The predictive model was based on the independent risk factors (multivariable logistic regression analysis, P <0.05). The results of the multivariable logistic regression model were then used to construct a nomogram using the packages “rms” and “DynNom” in the R Project software (version 4.2.1; http://www.r-project.org/). A regression model was used to calculate the score for each variable, and the predicted probability of HRPN was derived by summing each variable score.

### Nomogram performance

The predictive nomogram’s performance was assessed based on discrimination, calibration, and clinical utility. Discrimination is the ability of a model to correctly discriminate events from non-events. Receiver operating characteristic (ROC) curves were used to assess the discriminatory effectiveness of the predicted nomogram (21). The calibration measures how well the predicted probabilities match the true results. The Hosmer-Lemeshow test was used to assess the calibration ability, and a P-value >0.05 indicated satisfactory calibration (22).Calibration was then assessed further by constructing a nomogram calibration plot. Internal and external verifications were conducted using the bootstrapping method with 1000 repetitions (23). A decision curve analysis (DCA) was conducted to assess the clinical utility of the predictive nomograms based on the net benefit at different probability thresholds (24).

### Statistical analysis

Continuous normally distributed variables were compared using at a t-test, expressed as mean ± standard deviation (SD). If continuous variables are not normally distributed, the data are expressed as medians (interquartile range [IQR]) and compared between groups using the Mann-Whitney U test. Compared of categorical variables were performed using Pearson’s chi-squared or Fisher’s exact test. A two-sided P value of <0.05 was used for statistical significance. The SPSS software (v25.0; IBM Corp., Armonk, NY, USA) and the R Project software (v4.2.1; http://www.R-project.org) were used for the data analysis.

## Results

### Patient characteristics

**Figure 2:**
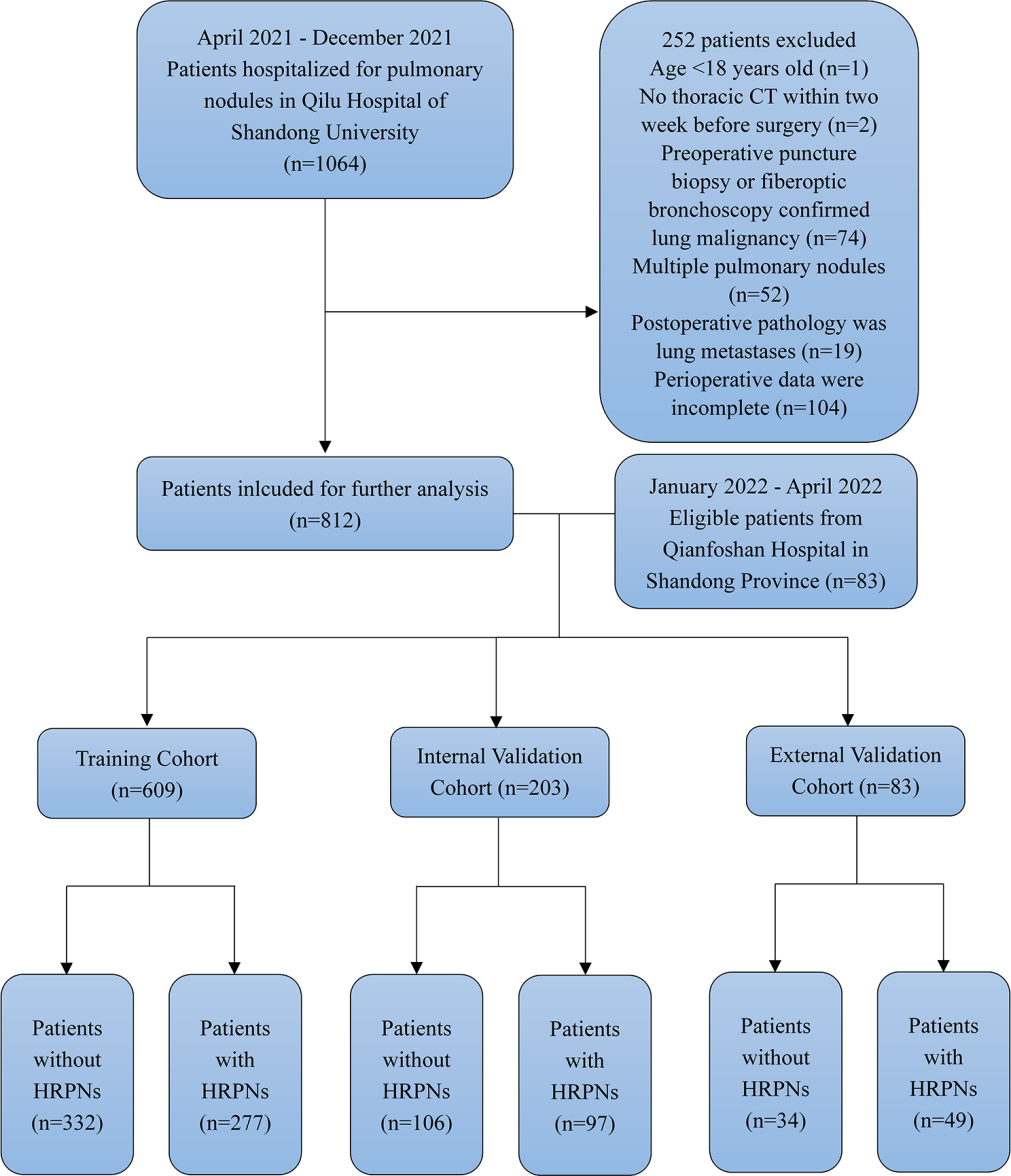
Flow diagram of patient selection through the study. HRPNs, High-risk pulmonary nodules.

The identification and selection process for eligible patients is shown in **Figure 2**. Overall, 895 patients with an HRPN incidence rate of 47.3% were included (423/895). The proportion of patients with IAC was 51.7% (463/895), while the proportion of patients with MIA was 24.5% (219/895) and the proportion of patients with AIS was 17.8% (159/895). Subsequently, 812 patients from Qilu Hospital of Shandong University were randomly assigned in a 3:1 ratio to the training (n = 609) or internal validation cohort (n = 203). Patients from Qianfoshan Hospital of Shandong Province were included in the independent external validation cohort (n = 83). No variables differed significantly between the training and validation cohorts (**Table 1**). Patients were divided into HRPNs and non-HRPN groups based on the presence or absence of HRPNs. The characteristics of the training and validation cohorts are presented in **Table 2**.

**Table 1:** Baseline characteristics of included patients and comparison between groups.

Characteristics	All	TC	IVC	EVC	P value	
	(n=812)	(n=609)	(n=203)	(n=83)	TC vs IVC	TC vs EVC
Age (years), median (IQR)	58.5 (52-66)	58 (52-66)	59 (51-67)	59 (51-66)	0.854	0.815
Gender, N(%)					0.422	0.015
Female	521 (64.16)	396 (65.02)	125 (61.58)	42 (50.60)		
Male	291 (35.84)	213 (34.98)	78 (38.42)	41 (49.40)		
BMI (kg/m^2^), median (IQR)	25.0 (22.9-27.2)	24.9 (22.9-27.1)	25.2 (23.0-27.6)	24.6 (23.1-26.7)	0.318	0.795
Smoking history, N(%)					0.201	0.065
(-)	636 (78.33)	484 (79.47)	152 (74.88)	58 (69.88)		
(+)	176 (21.67)	125 (20.53)	51 (25.12)	25 (30.12)		
CLD, N(%)					0.388	0.259
(-)	651 (80.17)	493 (80.95)	158 (77.83)	72 (86.75)		
(+)	161 (19.83)	116 (19.05)	45 (22.17)	11 (13.25)		
PF, N(%)					0.266	0.437
Normal	477 (58.74)	365 (59.93)	112 (55.17)	54 (65.06)		
Abnormal	335 (41.26)	244 (40.07)	91 (44.83)	29 (34.94)		
Family history of cancer, N(%)					0.315	0.060
(-)	667 (82.14)	495 (81.28)	172 (84.73)	75 (90.36)		
(+)	145 (17.86)	114 (18.72)	31 (15.27)	8 (9.64)		
Nodule diameter (d), N(%)					0.615	0.165
5 mm ≤ d ≤ 10 mm	229 (28.20)	177 (29.07)	52 (25.62)	22 (26.51)		
10 mm < d ≤ 20 mm	404 (49.75)	298 (48.93)	106 (52.22)	35 (42.17)		
20 mm < d ≤ 30 mm	179 (22.05)	134 (22.00)	45 (22.16)	26 (31.32)		
CTR, median (IQR)	0.21 (0.00-0.76)	0.20 (0.00-0.74)	0.24 (0.00-0.82)	0.22 (0.00-0.64)	0.189	0.666
CT value (n)					0.448	0.606
-800HU<n≤-600HU	217 (26.73)	166 (27.26)	51 (25.12)	21 (25.30)		
-600HU<n≤-400HU	245 (30.17)	190 (31.20)	55 (27.10)	28 (33.73)		
-400HU<n≤-200HU	143 (17.61)	105 (17.24)	38 (18.72)	18 (21.69)		
n>-200HU	207 (25.49)	148 (24.30)	59 (29.06)	16 (19.28)		
BEAS or Cavity Sign, N(%)					0.219	0.177
(-)	671 (82.64)	497 (81.61)	174 (85.71)	62 (74.70)		
(+)	141 (17.36)	112 (18.39)	29 (14.29)	21 (25.30)		
Lobulation, N(%)					0.571	0.416
(-)	555 (68.35)	420 (68.97)	135 (66.50)	53 (63.86)		
(+)	257 (31.65)	189 (31.03)	68 (33.50)	30 (36.14)		
Spiculation, N(%)					0.372	0.085
(-)	428 (52.71)	315 (51.72)	113 (55.67)	34 (40.96)		
(+)	384 (47.29)	294 (48.28)	90 (44.33)	49 (59.04)		
PT, N(%)					0.902	0.327
(-)	461 (56.77)	347 (56.98)	114 (56.16)	42 (50.60)		
(+)	351 (43.23)	262 (43.02)	89 (43.84)	41 (49.40)		
VC, N(%)					0.491	0.061
(-)	597 (73.52)	452 (74.22)	145 (71.43)	70 (84.34)		
(+)	215 (26.48)	157 (25.78)	58 (28.57)	13 (15.66)		
CEA (ng/ml), median (IQR)	2.23 (1.45-3.36)	2.22 (1.47-3.37)	2.25 (1.40-3.34)	2.35 (1.56-3.81)	0.961	0.264
CYFRA21-2 (ng/ml), median (IQR)	1.98 (1.44-2.57)	1.97 (1.44-2.53)	2.01 (1.41-2.65)	2.27 (1.60-3.35)	0.967	0.004
SCC (ng/ml), median (IQR)	0.96 (0.72-1.34)	0.94 (0.70-1.33)	1.01 (0.76-1.35)	0.80 (0.60-1.10)	0.248	0.003
Pro-GRP (pg/ml), median (IQR)	42.00 (34.10-51.08)	42.00 (34.05-51.88)	42.56 (34.33-49.70)	34.05 (27.26-41.40)	0.918	<.001
NSE (ng/ml), median (IQR)	19.00 (15.90-23.28)	19.00 (16.00-23.40)	19.10 (15.70-23.10)	14.10 (9.31-18.20)	0.942	<.001
High-risk pulmonary nodule, N(%)					0.626	0.028
(-)	438 (53.94)	332 (54.52)	106 (52.22)	34 (40.96)		
(+)	374 (46.06)	277 (45.48)	97 (47.78)	49 (59.04)		

^*^ P-value for the comparison between training cohort and validation cohort (internal validation cohort and external validation cohort).

IQR, interquartile range; (-), No; (+), Yes; TC, training cohort; IVC, internal validation cohort; EVC, external validation cohort; BMI, body mass index; CLD, chronic lung disease; PF, pulmonary function; CTR, consolidation tumor ratio; BEAS, bronchus encapsulated air sign; PT, pleural traction; VC, vascular convergence; CEA, carcinoembryonic antigen; CYFRA21-1, cytokeratin fragment antigen 21-1; SCC, squamous cell carcinoma antigen; Pro-GRP, progastrin-releasing peptide; NSE, neuron specific enolase.

**Table 2:** Clinical characteristics of patients with or without HRPN in training cohort and validation cohort (internal and external).

Characteristics	Training Cohort			Internal Validation Cohort			External Validation Cohort		
	Non HRPN (n=332)	HRPN (n=277)	P	Non HRPN (n=106)	HRPN (n=97)	P	Non HRPN (n=34)	HRPN (n=49)	P
Age^a^ (years)	56 (49-65)	62 (55-67)	<.001	55 (47.75-63)	63 (56-69)	<.001	56.5 (47.75-64)	61 (54.5-67.5)	0.046
Gender^b^			0.058			0.012			0.090
Female	227 (68.37)	169 (61.01)		74 (69.81)	51 (52.58)		21 (61.76)	21 (42.86)	
Male	105 (31.63)	108 (38.99)		32 (30.19)	46 (47.42)		13 (38.24)	28 (57.14)	
BMI^a^ (kg/m^2^)	24.7 (22.7-27.0)	25.2 (23.2-27.2)	0.077	25.0 (22.4-27.2)	25.3 (23.6-28.0)	0.184	24.7 (23.0-26.5)	24.6 (23.1-26.8)	0.732
Smoking history^b^			0.008			<.001			0.011
(-)	277 (83.43)	207 (74.73)		90 (84.91)	62 (63.92)		29 (85.29)	29 (59.18)	
(+)	55 (16.57)	70 (25.27)		16 (15.09)	35 (36.08)		5 (14.71)	20 (40.82)	
CLD^b^			0.011			0.001			0.739
(-)	281 (84.64)	212 (76.53)		92 (86.79)	66 (68.04)		30 (88.24)	42 (85.71)	
(+)	51 (15.36)	65 (23.47)		14 (13.21)	31 (31.96)		4 (11.76)	7 (14.29)	
PF^b^			0.013			<.001			0.681
Normal	214 (64.46)	151 (54.51)		71 (66.98)	41 (42.27)		23 (67.65)	31 (63.27)	
Abnormal	118 (35.54)	126 (45.49)		35 (33.02)	56 (57.73)		11 (32.35)	18 (36.73)	
Family history of cancer^b^			0.811			0.479			0.085
(-)	271 (81.63)	224 (80.87)		88 (83.02)	84 (86.60)		33 (97.06)	42 (85.71)	
(+)	61 (18.37)	53 (19.13)		18 (16.98)	13 (13.40)		1 (2.94)	7 (14.29)	
Nodule diameter^b^ (d)			<.001			<.001			<.001
5 mm ≤ d ≤ 10 mm	152 (45.78)	25 (9.02)		47 (44.34)	5 (5.15)		15 (44.12)	7 (14.28)	
10 mm < d ≤ 20 mm	150 (45.18)	148 (53.43)		53 (50.00)	53 (54.64)		15 (44.12)	20 (40.82)	
20 mm < d ≤ 30 mm	30 (9.04)	104 (37.55)		6 (5.66)	39 (40.21)		4 (11.76)	22 (44.90)	
CTR^a^	0.00 (0.00-0.14)	0.71 (0.27-1.00)	<.001	0.00 (0.00-0.00)	0.82 (0.41-1.00)	<.001	0.00 (0.00-0.13)	0.51 (0.18-0.77)	<.001
CT value^b^ (n)			<.001			<.001			<.001
-800HU<n≤-600HU	158 (47.59)	8 (2.89)		50(47.17)	1(1.03)		15 (44.12)	6 (12.25)	
-600HU<n≤-400HU	129 (38.86)	61 (22.02)		41(38.68)	14(14.43)		14 (41.18)	14 (28.57)	
-400HU<n≤-200HU	28 (8.43)	77 (27.80)		12(11.32)	26(26.81)		4 (11.76)	14 (28.57)	
n>-200HU	17 (5.12)	131 (47.29)		3(2.83)	56(57.73)		1 (2.94)	15 (30.61)	
BEAS or Cavity Sign^b^			0.020			<.001			0.064
(-)	282 (84.94)	215 (77.62)		101(95.28)	73(75.26)		29 (85.29)	33 (67.35)	
(+)	50 (15.06)	62 (22.38)		5(4.72)	24(24.74)		5 (14.71)	16 (32.65)	
Lobulation^b^			<.001			<.001			0.014
(-)	286 (86.14)	134 (48.38)		95(89.62)	40(41.24)		27 (79.41)	26 (53.06)	
(+)	46 (13.86)	143 (51.62)		11(10.38)	57(58.76)		7 (20.59)	23 (46.94)	
Spiculation^b^			<.001			<.001			<.001
(-)	219 (65.96)	96 (34.66)		80(75.47)	33(34.02)		23 (67.65)	11 (22.45)	
(+)	113 (34.04)	181 (65.34)		26(24.53)	64(65.98)		11 (32.35)	38 (77.55)	
PT^b^			<.001			<.001			0.032
(-)	227 (68.37)	120 (43.32)		83(78.30)	31(31.96)		22 (64.71)	20 (40.82)	
(+)	105 (31.63)	157 (56.68)		23(21.70)	66(68.04)		12 (35.29)	29 (59.18)	
VC^b^			0.001			0.689			0.416
(-)	264 (79.52)	188 (67.87)		77(72.64)	68(70.10)		30 (88.24)	40 (81.63)	
(+)	68 (20.48)	89 (32.13)		29(27.36)	29(29.90)		4 (11.76)	9 (18.37)	
CEA^a^ (ng/ml)	1.93 (1.31-2.98)	2.49 (1.72-4.05)	<.001	1.76 (1.25-2.74)	2.74 (1.83-4.21)	<.001	2.12 (1.11-3.93)	2.46 (1.66-3.74)	0.418
CYFRA21-2^a^ (ng/ml)	1.83 (1.38-2.45)	2.05 (1.55-2.72)	0.001	1.87 (1.34-2.65)	2.13 (1.54-2.66)	0.122	2.23 (1.30-3.32)	2.27 (1.81-3.46)	0.350
SCC^a^ (ng/ml)	0.92 (0.72-1.29)	0.98 (0.69-1.36)	0.414	1.07 (0.80-1.37)	0.90 (0.72-1.30)	0.067	0.80 (0.60-1.13)	0.80 (0.60-1.05)	0.503
Pro-GRP^a^ (pg/ml)	42.39	41.77	0.901	41.14	43.03	0.064	32.00	34.24	0.846
	(34.83-50.54)	(33.52-53.17)		(32.07-49.16)	(36.49-51.05)		(27.62-41.37)	(26.95-42.45)	
NSE^a^ (ng/ml)	19.00	19.00	0.899	19.50	19.00	0.961	13.62	15.20	0.245
	(15.83-23.48)	(16.05-23.15)		(15.35-24.08)	(16.00-22.45)		(8.91-16.20)	(10.18-19.60)	

a Variables were compared by the Mann-Whitney U test between groups, and data were expressed as medians (interquartile range [IQR])

b Variables were compared using Pearson's chi-square test or Fisher's exact test, and results were expressed as percentages.

HRPN, high-risk pulmonary nodule; (-), No; (+), Yes; BMI, body mass index; CLD, chronic lung disease; PF, pulmonary function; CTR, consolidation tumor ratio; BEAS, bronchus encapsulated air sign; PT, pleural traction; VC, vascular convergence; CEA, carcinoembryonic antigen; CYFRA21-1, cytokeratin fragment antigen 21-1; SCC, squamous cell carcinoma antigen; Pro-GRP, progastrin-releasing peptide; NSE, neuron specific enolase.

### Identification of HRPN risk factors

Univariable and multivariable logistic regression analyses were performed on the training cohort to identify HRPN risk factors (**Table 3**). Univariable logistic regression analysis showed that age, gender, body mass index, smoking history, chronic lung disease, abnormal pulmonary function, size, CTR, and CT values of PNs, BEAS or cavity sign, lobulation, spiculation, pleural traction, vascular convergence, and blood tumor marker (CEA, CYFRA21-1, SCC, Pro-GRP, and NSE) levels were potential risk factors (P< 0.2). Further multivariable logistic regression analysis (forward stepwise regression) showed that the PN size (P<0.001), CTR (odds ratio [OR] = 3.8338; 95% confidence interval [CI]: 1.661-8.868; P=0.002), CT value of PNs (P<0.001), and blood CEA levels (OR = 1.701; 95% CI: 1.702–2.701; P =0.024) were independently associated with HRPNs. For some continuous variables in the study factors (CTR, CEA, CYFRA21-1, SCC, Pro- GRP, and NSE), their optimal cutoff values were determined by plotting their ROC curves. The best cutoff value was used as the standard for converting into binary variables and included in the regression analysis (**Supplementary Table 1**).

**Table 3:** Univariable and multivariable logistic regression analysis of risk factors for HRPN in the training cohort.

Variable	Univariable analysis			Multivariable analysis		
	OR	95%CI	P	OR	95%CI	P
Age (years)	1.049	1.031-1.067	<.001			
Gender			0.058			
Female	Ref.					
Male	1.382	0.989-1.930				
BMI (kg/m^2^)	1.039	0.991-1.090	0.115			
Smoking history			0.008			
(-)	Ref.					
(+)	1.703	1.146-2.532				
CLD			0.012			
(-)	Ref.					
(+)	1.689	1.124-2.540				
PF			0.013			
Normal	Ref.					
Abnormal	1.513	1.092-2.097				
Family history of cancer			0.811			
(-)	Ref.					
(+)	1.051	0.699-1.581				
Nodule diameter (d)			<.001			0.001
5 mm ≤ d ≤ 10 mm	Ref.			Ref.		
10 mm < d ≤ 20 mm	5.999	3.712-9.696		2.234	1.242-4.017	
20 mm < d ≤ 30 mm	21.077	11.725-37.888		3.873	1.888-7.944	
CTR			<.001			0.002
≤0.265	Ref			Ref.		
>0.265	21.642	14.154-33.092		3.838	1.661-8.868	
CT value (n)			<.001			<.001
-800HU<n≤-600HU	Ref.			Ref.		
-600HU<n≤-400HU	9.339	4.312-20.227		7.287	3.308-16.048	
-400HU<n≤-200HU	54.312	23.645-124.757		11.846	3.962-35.422	
n>-200HU	152.191	63.655-363.870		23.553	6.909-80.289	
BEAS or Cavity Sign			0.021			
(-)	Ref.					
(+)	1.626	1.077-2.457				
Lobulation			<.001			
(-)	Ref.					
(+)	6.635	4.490-9.804				
Spiculation			<.001			
(-)	Ref.					
(+)	3.654	2.612-5.112				
PT			<.001			
(-)	Ref.					
(+)	2.828	2.030-3.941				
VC			0.001			
(-)	Ref.					
(+)	1.838	1.273-2.653				
CEA			<.001			0.024
≤1.965 ng/ml	Ref.			Ref.		
>1.965 ng/ml	2.399	1.718-3.350		1.701	1.072-2.701	
CYFRA21-2			<.001			
≤1.785 ng/ml	Ref.					
>1.785 ng/ml	1.863	1.340-2.590				
SCC			0.015			
≤1.655 ng/ml	Ref.					
>1.655 ng/ml	1.850	1.126-3.039				
Pro-GRP			0.078			
≤33.455 pg/ml	Ref.					
>33.455 pg/ml	0.706	0.480-1.039				
NSE			0.180			
≤17.75 ng/ml	Ref.					
>17.75 ng/ml	1.251	0.902-1.735				

HRPN, high-risk pulmonary nodule; (-), No; (+), Yes; BMI, body mass index; CLD, chronic lung disease; PF, pulmonary function; CTR, consolidation tumor ratio; BEAS, bronchus encapsulated air sign; PT, pleural traction; VC, vascular convergence; CEA, carcinoembryonic antigen; CYFRA21-1, cytokeratin fragment antigen 21-1; SCC, squamous cell carcinoma antigen; Pro-GRP, progastrin-releasing peptide; NSE, neuron specific enolase.

### Nomogram construction

**Figure 3:**
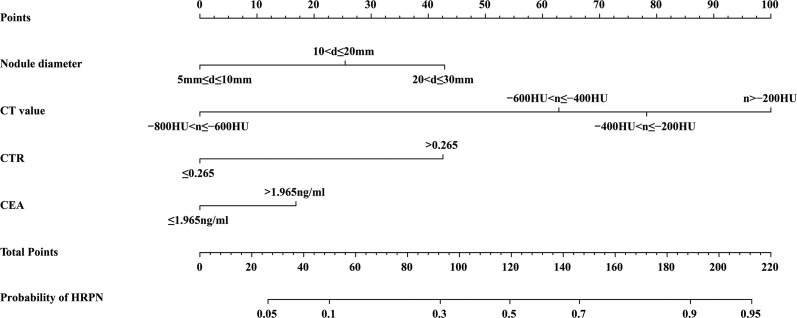
A nomogram predicting the probability that PNs with diameter ≤3 cm are HRPNs. Draw a vertical line from each variable’s corresponding axis to the point axis to get the points for that variable. Summing the scores for each variable to obtain the total score, the probability of predicting HRPNs can be estimated by projecting the total score onto the lower total score axis. PNs, Pulmonary nodules; HRPNs, High-risk pulmonary nodules.

Four independent HRPN risk factors were included in the logistic regression models. A nomogram of predicted HRPNs based on the coefficients from the multiple logistic regression model was plotted using the “rms” package in the R statistical software (**Figure 3**). The nomogram shows seven axes, with axes 2-5 representing the four variables in the predictive model. Each variable was scored on a numeric scale ranging from 0 to 100. A total score can be calculated by summing each factor’s score. By projecting the total score onto the lower total score scale axis, we can predict the likelihood of HRPNs. Furthermore, in order to facilitate the widespread use of our predictive nomograms among thoracic surgeons, we built an operator interface on a web page using the “Dynnom” package to calculate the predicted probabilities of HRPNs. By selecting the patient’s preoperative imaging features and tumor marker levels, the user can obtain the predicted probability that PN is a high-risk type.

### Predictive performance and nomogram validation

**Figure 4:**
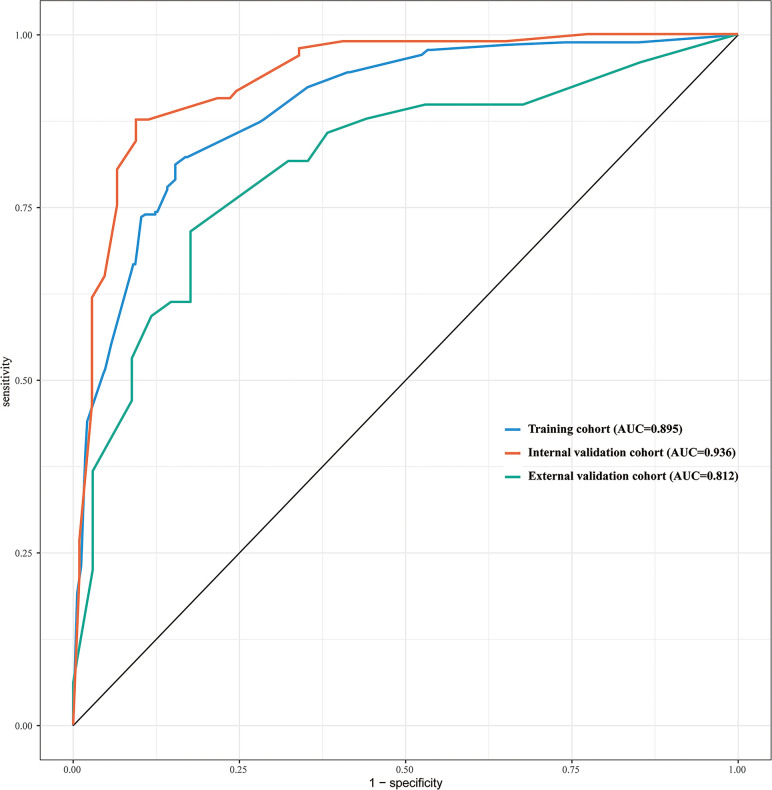
ROC curves of the nomogram used to predict the probability of HRPNs in training and validation cohorts. ROC, Receiver operating characteristics; HRPNs, High-risk pulmonary nodules; AUC, Area under the ROC curve.

**Figure 5:**
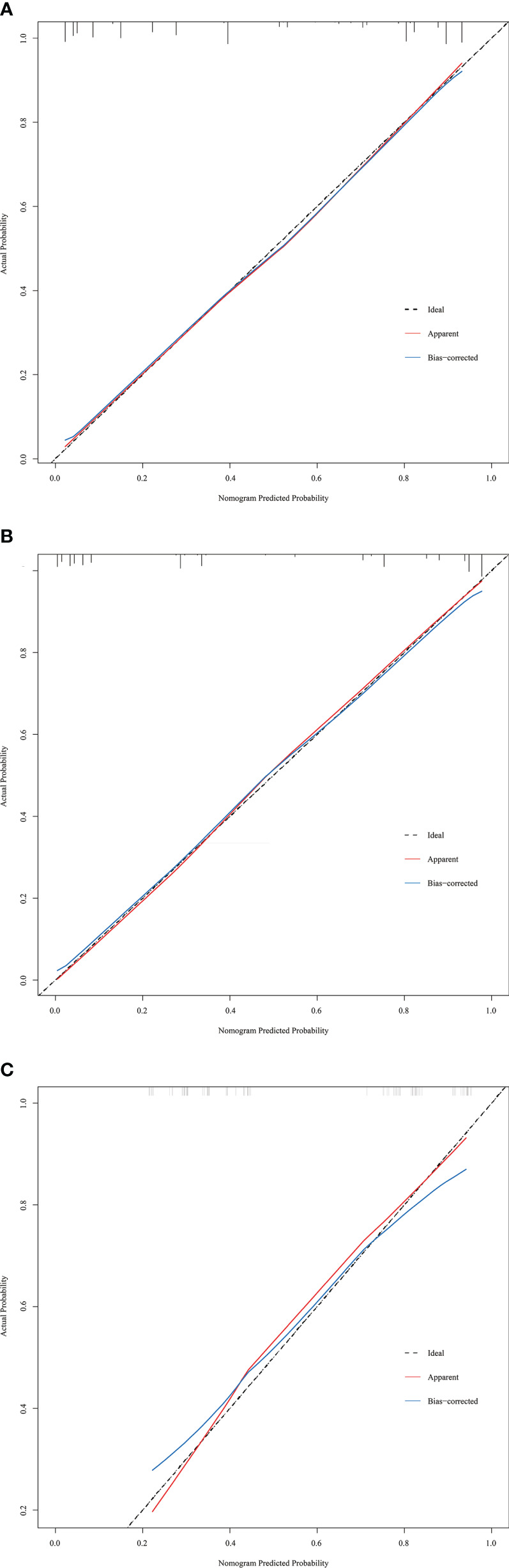
Calibration curves of the prediction nomogram in the training cohort **(A)**, internal validation cohort **(B)** and external validation cohort **(C)**. The x-axis represents the nomogram-predicted probability, and the y-axis represents the actual probability of HRPNs. The black pointed line represents the ideal curve, the red solid line represents the apparent curve (non-correction), and the blue solid line represents the bias-correction curve by bootstrapping (B = 1000 repetitions). HRPNs, High-risk pulmonary nodules.

The discriminative ability of the prediction model and nomogram was assessed using an ROC curve (**Figure 4**). The area under the ROC curve (AUC) was 0.895 (95% CI: 0.870-0.920), 0.936 (95% CI:0.903-0.970), and 0.812 (95% CI:0.717-0.906) for the training, internal validation, and external validation cohorts, respectively. This indicated that the nomogram’s prediction accuracy was relatively good. For the predicted probability of HRPNs, the optimal cut-off value was approximately 45.09%, with a sensitivity and specificity of 0.846 and 0.812, respectively (**Supplementary Table 1**). The Hosmer-Lemeshow test showed excellent calibration ability (P=0.418, 0.916, and 0.975 in the training, internal validation, and external validation cohorts, respectively). The nomograph’s calibration curve of HRPN predicted probabilities also showed excellent concordance between the predicted and actual results (**Figure 5**).

### Predictive nomogram’s clinical utility

**Figure 6:**
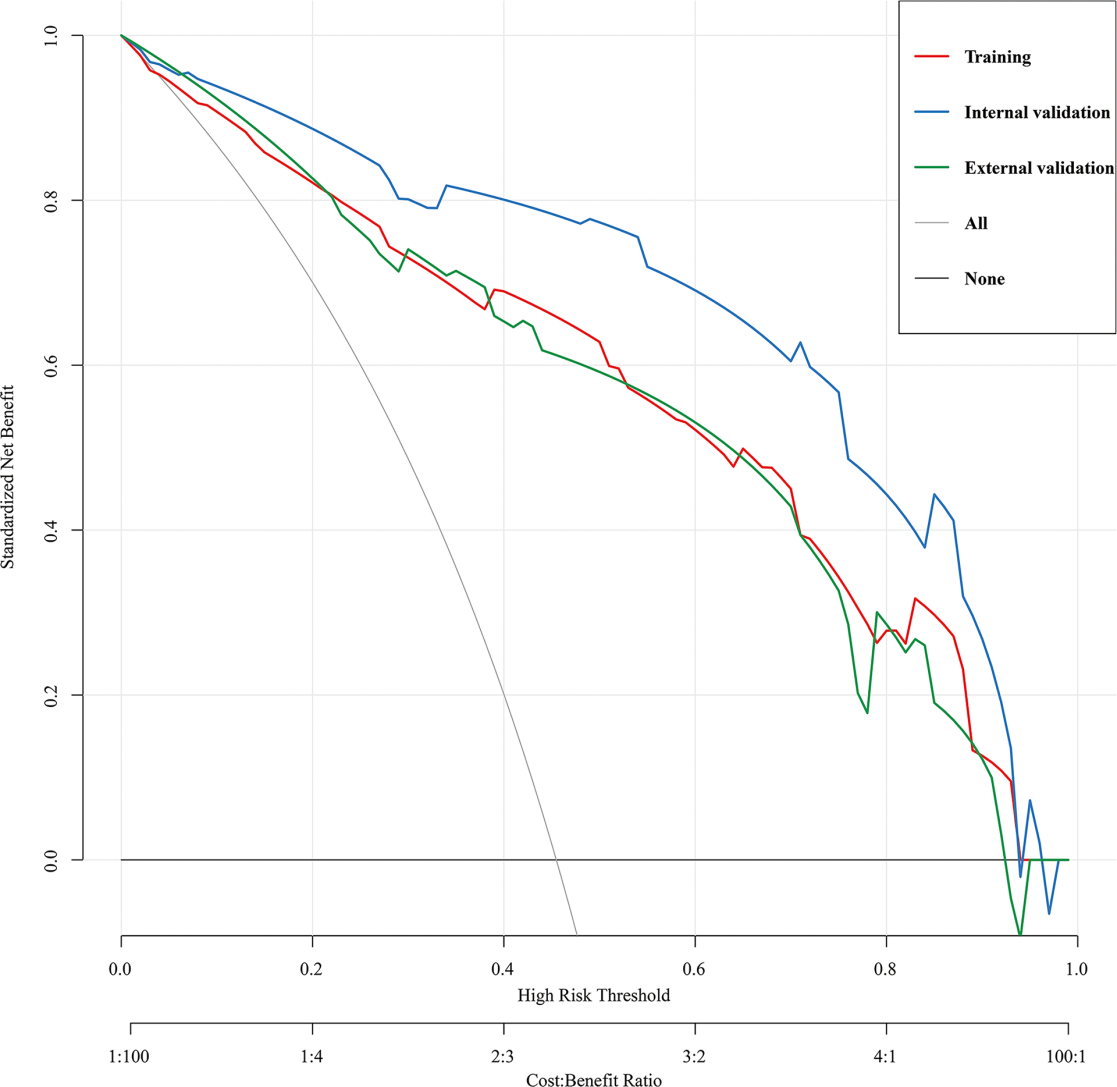
Decision curve analysis for the HRPNs nomogram in the training and validation cohorts. The y-axis measures the net benefit, the black line represents the assumption of HRPNs-none-patients, the gray line represents the assumption of HRPNs-all-patients, the red line represents the training cohort, the blue line represents the internal validation cohort, the green lines represents the external validation cohort. HRPNs, High-risk pulmonary nodules.

The predictive nomogram’s clinical utility was assessed using DCA(**Figure 6**). The results showed that the nomogram for HRPNs’ prediction provided a larger net gain with a broader range of threshold probabilities for predicting HRPNs risk across both the training and validation cohorts. It also proved evidence that the nomogram can be applied clinically and assist surgeons in making better clinical decisions.

## Discussion

With the popularity of lung cancer screening using low-dose thoracic thin-slice CT, the PN detection rate has increased (2). For mixed ground-glass nodules, it is difficult to make a definitive preoperative histopathological prediction of PN based on its imaging features (such as lobulation, spiculation, or pleural stretch) (25). Consequently, many low-grade PNs are overtreated, leading to increased hospitalization costs, longer hospital stays, and a higher risk of postoperative complications (26). Therefore, in this study, we combined patients’ preoperative imaging information and blood tumor marker levels to develop a clinical prediction model and designed a nomogram with good predictive performance for the degree of PNs risk. Clinicians can estimate the probability that a patient’s PN is HRPN before surgery using this predictive nomogram, thereby making sound treatment decisions for PNs with various risks.

This study showed that PN diameter, CT value, CTR, and blood CEA level were independent risk factors for HRPNs. One of the most important imaging features for determining the malignancy of PNs is their diameter. As the diameter of the nodule increases, the depth of invasion and the probability of adverse PN pathological types also increase. Our study showed that PNs with diameters of 10-20 mm and 20-30 mm had a 2.234- fold and 3.873-fold higher risk of HRPNs, respectively compared with PNs of 5–10 mm. This finding is consistent with that of previous studies on the size and malignancy of PNs (25, 27). Previous studies have demonstrated that the average CT value of PNs can discriminate between a variety of invasive lung cancers, and a higher CT value indicates a higher possibility of malignancy (28, 29). In one study, it was found that the average CT value could be used to predict the growth of pure ground-glass nodules with an optimum cut-off value of -670HU (30). In another study by Ikeda et al., it was proven that a cut-off value of -584 HU was helpful in distinguishing AAH from AIS and of -472 HU was helpful in distinguishing AIS from IAC (31). Koezuka et al. also confirmed that CT values reflect the cellular structure and density of the lung nodules (32). The highly invasive component was usually present in the site with a high CT value, while the site with a low CT value was diagnosed as having lower invasiveness based on the final pathological result. According to the results of Ikeda, Eguchi and Koezuka et al., the CT value of PNs was divided by a gradient of 200HU, which could well reflect the change degree of the solid component of PNs (30–32). Therefore, our prediction model was divided by this gradient. The final results demonstrated that the higher the CT value of the nodule, the higher the risk of HRPNs (OR=7.287, 11.846, and 23.553, respectively) compared with PNs with CT values between -800HU and -600HU. These results are in agreement with those of Ikeda et al. and Eguchi et al (30, 31).

The study of CTR has a long history, and the choice of the optimal cutoff value is still controversial. Suzuki et al. have shown that a CTR ≤0.25 is one of the radiological criteria for non-invasive lung cancer, and the specificity of judging the nodule malignancy before surgery can reach 98.7% (18). According to literature reports, mixed ground-glass nodules with CTR ≤0.5 and diameter ≤1cm have better 5-year OS and recurrence-free survival (33, 34). A study from Japan showed that PNs with a CTR <0.5 and a diameter of less than 2 cm had better recurrence-free survival and OS. And CTR >0.5 often predicted a poor prognosis for patients with these PNs (35). In this study, after multivariable logistic regression analysis, CTR was established as an independent risk factor for HRPNs (OR=3.838, P=0.002). ROC curve analysis was performed on the CTR of the included patients, with an AUC value of 0.869 and an optimal cut-off value of 0.265 (similar to the study of Suzuki K et al.) (18). This indicates that the CTR has a good predictive performance for HRPNs. However, the optimal cutoff value was different from that reported in Sun’s study (34), which may be due to the different diameter criteria of the included PNs. In addition, unlike Sun’s study, PNs were reclassified in this study using the fifth edition of the World Health Organization classification of thoracic tumors. This will undoubtedly affect the optimal cut-off value for the CTR.

Regarding tumor markers, we confirmed the diagnostic value of CEA in predicting the risk of HRPNs. Yuan et al. demonstrated that the sensitivities of CEA, CYFRA21-1, and NSE for diagnosing lung cancer were 56.5%, 56.1%, and 19.1%, respectively (16). Molina et al. reported that the diagnostic sensitivity and specificity could reach 88.5% and 82%, respectively, combined with six hematological tumor markers (CA153, CEA, CYFRA21- 1, NSE, Pro-GRP, and SCC) (36). After univariable and multivariable logistic regression analysis of blood tumor markers (CEA, CYFRA21-1, SCC, NSE, and Pro-GRP), only CEA was an independent factor for HRPNs (OR=1.701, 95% CI: 1.072-2.701, P=0.024). This may be due to the high sensitivity of some tumor markers to certain pathological lung cancer types. For example, CYFRA21-1 and SCC are more sensitive to lung squamous cell carcinoma (37), while Pro-GRP and NSE are more sensitive to small cell lung carcinoma (38). Blood CEA levels can be used as a combined index to predict the occurrence of lung cancer, which has good universality. ROC analysis of blood CEA levels in the included patients showed an AUC value of 0.641 with an optimal cutoff value of 1.965 ng/mL (similar to that reported in the study by Zheng et al), indicating that blood CEA levels have better predictive performance for HRPNs than other tumor markers.

It is noteworthy that the multivariable logistic regression analysis showed that the imaging features of lung cancer (lobulation, spiculation, BEAS or cavity sign pleural traction, and vascular convergence) were not independent risk factors for HRPNs, in contrast with previous research findings (39, 40). Based on our analysis, we believe that the imaging features of lung cancer have good sensitivity and specificity for the benign and malignant judgment of PNs; however, it is difficult to use these indicators for evaluating the malignant degree of PNs. This is because, regardless of whether the degree of malignancy is high or low, the above-mentioned imaging features may appear as malignant PNs, which will undoubtedly reduce the accuracy of the judgment of these indicators. According to Liang and Feng’s study, pulmonary nodules with final pathology of benign lesions, precancerous lesions, and lung adenocarcinoma can exhibit spiculation on the thoracic CT sign (41, 42). This infers subtly that the imaging characteristics of lung cancer have a low degree of specificity.

Several models for predicting benign and malignant PNs have been reported (40, 43); however, no model has focused on predicting the degree of PN malignancy. The advantages of our method over previously published predictive models are as follows: First, we visualized this predictive model, built a nomogram, and subsequently constructed an operating interface for our nomogram on the web page (http://lungnodules.shinyapps.io/Predict_QiuJH/), which greatly optimized the computational process and enhanced the practicality of using this model in clinical practice. Secondly, we included patients with PNs with a maximum diameter of ≤3 cm on imaging into the model as much as possible and built a preoperative risk prediction model to predict the degree of PN malignancy. Furthermore, this model can be applied to the majority of patients with PNs, greatly increasing its usability. Third, we developed a predictive model that used preoperative imaging features and blood CEA levels to predict the risk of developing HRPNs regardless of the patient’s final histopathological outcome. Preoperative non-surgical biopsy (such as CT-guided lung biopsy and bronchoscopy) can be used to obtain PN histopathology, their invasive nature and potential risks limits their clinical usefulness. Therefore, this model can predict the high-risk probability of PNs in patients before surgery to guide the selection of surgical methods to reduce surgical trauma and shorten patients’ hospitalization time. Fourth, we used CTR to predict the degree of PN malignancy, which has not been observed in previous studies. Furthermore, we employed internal- external validation in model validation, leveraging patient data from both centers to validate our model. Final validation results also demonstrated the good prediction accuracy of our model. Lastly, we utilized DCA to assess the clinical utility of predictive nomograms. Based on the decision curve, the nomogram model had a clear net benefit (HRPN incidence across all cohorts in this study) in the threshold range of 45-60%, suggesting that the nomogram has a high utility in the clinic.

The clinical predictive nomogram constructed can help thoracic surgeons use preoperative imaging and tumor marker features to assess the probability of HRPNs preoperatively to make better clinical decisions. For patients with HRPNs, standard lobectomy and systematic lymph node dissection can be used to reduce the recurrence rate of lung cancer after surgery, while sublobar resection (lung wedge resection and anatomic segmental lung resection) can be performed in patients with LRPNs to avoid overtreatment and better protect lung function. Related studies from Japan have shown that segmentectomy can be utilized as the standard treatment for PNs with CTR < 0.5 and tumor diameter < 3cm. Additionally, for pulmonary nodules with CTR > 0.5, the recurrence-free survival and disease-free survival of patients were improved by lobectomy. This outcome matched the predictions made by our prediction model (35, 44). In our hospital, after preoperative evaluation of the patient’s PNs, we performed surgical treatment according to the above treatment strategies, which ensured precise patient treatment, reduced the hospitalization time and patient costs, and realized lung cancer-enhanced recovery strategies.

There are several limitations to the present study. First, this retrospective study may limit the generalizability of our predictive nomogram, and uncontrolled confounding factors may also arise. Furthermore, this predictive model has undergone both internal and external validation; hence, selection biases present in the training cohort may be present in the internal validation cohort. Thus, multiple external validations in a more central setting are required to determine if the nomogram is suitable for widespread use in other populations. Finally, we did not include factors that may be related to HRPNs in our study, such as nodule doubling time, blood tissue polypeptide antigen level, and CA125 level. These factors were absent from our database and should be explored in future studies.

## Conclusion

Based on PNs preoperative imaging characteristics and blood tumor marker levels, a clinical nomogram to predict the probability of HRPNs was established, and a good prediction effect was achieved. The probability of HRPNs in patients with PNs can be assessed using this nomogram. Different treatment strategies can be applied to HRPNs and LRPNs to achieve precise treatment and accelerated patient recovery.

## Data availability statement

The raw data supporting the conclusions of this article will be made available by the authors, without undue reservation.

## Ethics statement

The studies involving human participants were reviewed and approved by The Ethics Committee of Qilu Hospital of Shandong University. The patients/participants provided their written informed consent to participate in this study.

## Author contributions

Conception and design: JQ and HT. Administrative support: HT and WY. Provision of study materials or patients: JQ, RL and XM. Collection and assembly of data: JQ, RL, YW, XM and BL. Data analysis and interpretation: JQ, RL and CQ. All authors contributed to the article and approved the submitted version.
