# Supplementary material for: A nomogram combining thoracic CT and tumor markers to predict the malignant grade of pulmonary nodules ≤3 cm in diameter
Source: Front Oncol. 2023 Jun 8;13:1196883. doi: 10.3389/fonc.2023.1196883 (PMC10285407; doi:10.3389/fonc.2023.1196883)
Supplement: Supplementary file 2 [file Presentation_1.pdf]

Supplementary Figure Legends

Supplementary Figure 1. (A) Lobulation: A 69-year-old woman presented with MIA of the right lower lobe confirmed by postoperative histology. (B) Spiculation: A 69-year-old woman presented with MPA in the right middle lobe confirmed by postoperative histology. (C) Bronchus-encapsulated air sign: A 49-year-old woman presented with AIS in the right upper lobe confirmed by postoperative histology. MIA = Minimally invasive adenocarcinoma; MPA = Micropapillary invasive adenocarcinoma; AIS = Adenocarcinoma in situ

Supplementary Figure 2. (A) Cavity signs: A 67-year-old man presented with MIA in the right upper lobe confirmed by postoperative histology. (B) Pleural traction: A 47-year-old woman presented with IAC in the left upper lobe confirmed by postoperative histology. (C) Vascular convergence: A 60-year-old woman presented with MIA in the left upper lobe confirmed by postoperative histology. MIA = Minimally invasive adenocarcinoma; IAC = Invasive adenocarcinoma
