# Supplementary figures and images for: A nomogram combining thoracic CT and tumor markers to predict the malignant grade of pulmonary nodules ≤3 cm in diameter
Source: Front Oncol. 2023 Jun 8;13:1196883. doi: 10.3389/fonc.2023.1196883 (PMC10285407; doi:10.3389/fonc.2023.1196883)

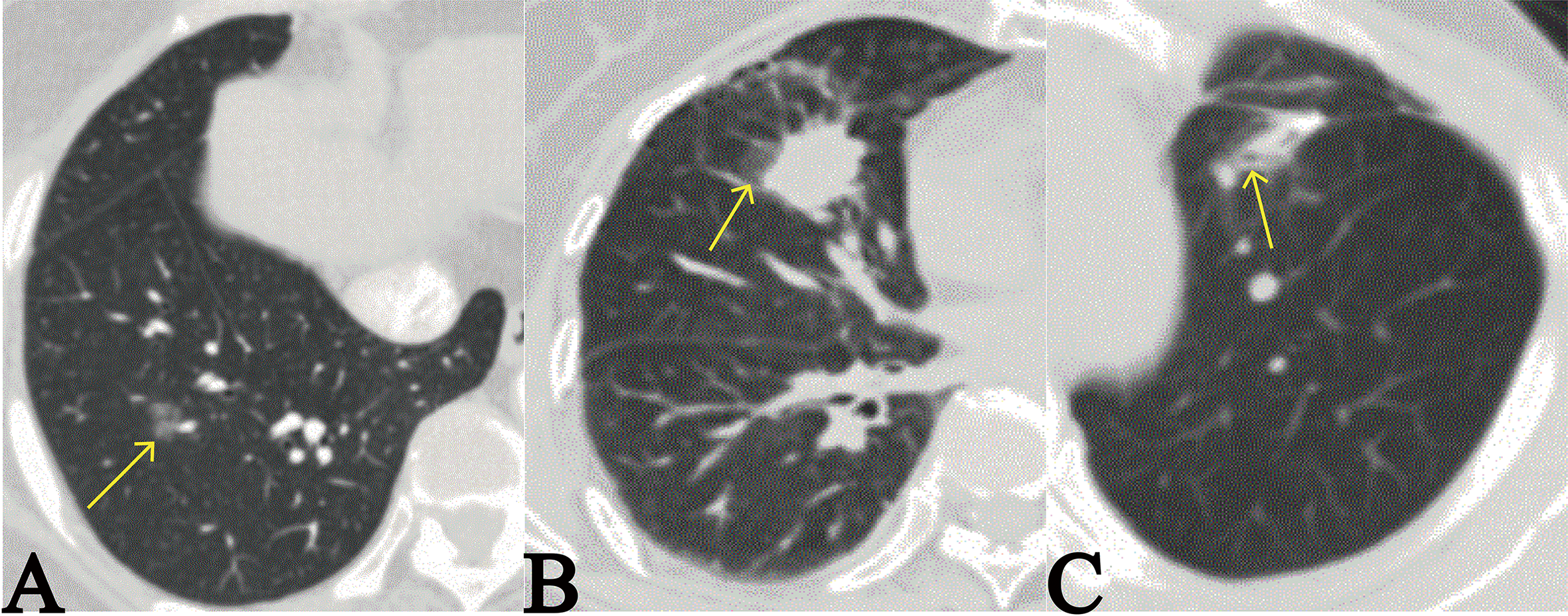

Supplement: Supplementary file 3 [file Image_1.tif]

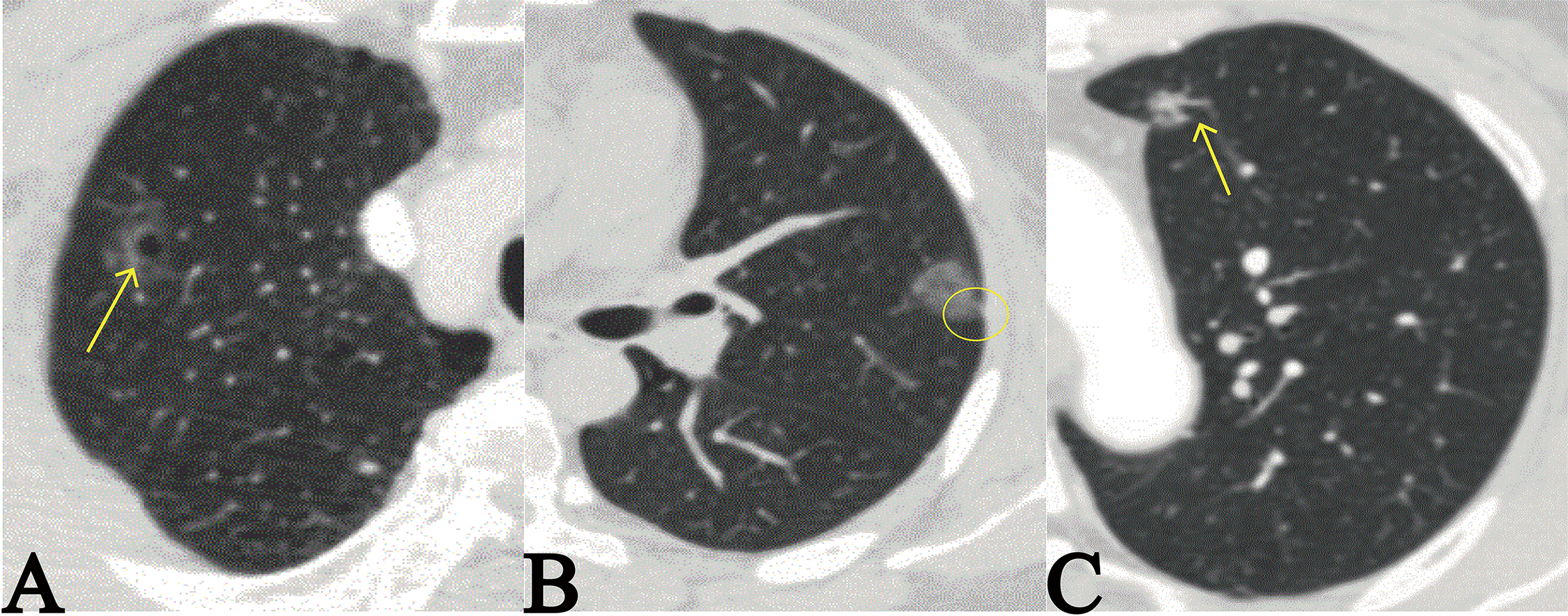

Supplement: Supplementary file 4 [file Image_2.tif]
