# Supplementary material for: A nomogram combining thoracic CT and tumor markers to predict the malignant grade of pulmonary nodules ≤3 cm in diameter
Source: Front Oncol. 2023 Jun 8;13:1196883. doi: 10.3389/fonc.2023.1196883 (PMC10285407; doi:10.3389/fonc.2023.1196883)
Supplement: Supplementary file 5 [file Table_1.docx]

**Supplementary Table 1.** Performance comparison of the ROC curves of CTR and blood tumor markers.

| Index | CTR | CEA | CYFRA21-1 | SCC | Pro-GRP | NSE |
| --- | --- | --- | --- | --- | --- | --- |
| AUC | 0.869 | 0.641 | 0.575 | 0.519 | 0.503 | 0.503 |
| Best Cut-off Value | 0.265 | 1.965 | 1.785 | 1.655 | 33.455 | 17.75 |
| Sensitivity | 75.81 | 69.31 | 66.46 | 15.52 | 24.91 | 63.18 |
| Specificity | 87.35 | 51.51 | 48.49 | 90.96 | 81.02 | 42.17 |
| Negative Predictive Value | 81.23 | 66.80 | 63.39 | 56.34 | 56.39 | 57.85 |
| Positive Predictive Value | 83.33 | 54.39 | 51.83 | 58.90 | 52.27 | 47.68 |
| True Positive Rate | 75.81 | 69.31 | 66.43 | 15.52 | 24.91 | 63.18 |
| False Positive Rate | 12.65 | 48.49 | 51.51 | 9.036 | 18.98 | 57.83 |
| True Negatice Rate | 87.35 | 51.51 | 48.49 | 90.96 | 81.02 | 42.17 |
| False Negative Rate | 24.19 | 30.69 | 33.57 | 84.48 | 75.09 | 36.82 |
| False Discovery Rate | 16.67 | 45.61 | 48.17 | 41.10 | 47.73 | 52.32 |
| Accuracy | 82.10 | 59.61 | 56.65 | 56.65 | 55.50 | 51.72 |
| Precision | 83.33 | 54.39 | 51.83 | 58.90 | 52.27 | 47.68 |
| Youden Index | 163.16 | 120.82 | 114.92 | 106.49 | 105.93 | 105.36 |

ROC= receiver operating characteristic; CTR= consolidation tumor ratio; AUC=area under curve; CEA=carcinoembryonic antigen; CYFRA21-1=cytokeratin fragment antigen 21-1; SCC=squamous cell carcinoma antigen; Pro-GRP=progastrin-releasing peptide; NSE=neuron specific enolase.
